# Alterations in brain morphology and functional connectivity mediate cognitive decline in carotid atherosclerotic stenosis

**DOI:** 10.3389/fnagi.2024.1395911

**Published:** 2024-06-20

**Authors:** Subinuer Maimaitiaili, Chen Tang, Cheng Liu, Xiaochen Lv, Zhipeng Chen, Mengqiang Zhang, Jing Cai, Zishun Liang, Biao Zhao, Wen Zhang, Tong Qiao

**Affiliations:** ^1^Department of Vascular Surgery, Nanjing Drum Tower Hospital, Affiliated Hospital of Medical School, Nanjing University, Nanjing, China; ^2^Department of Radiology, Nanjing Drum Tower Hospital, Affiliated Hospital of Medical School, Nanjing University, Nanjing, China

**Keywords:** maximum degree of carotid stenosis, cognitive impairment, gray matter volume, cortical thickness, functional connectivity

## Abstract

**Background:**

Patients with carotid atherosclerotic stenosis (CAS) often have varying degrees of cognitive decline. However, there is little evidence regarding how brain morphological and functional abnormalities impact the cognitive decline in CAS patients. This study aimed to determine how the brain morphological and functional changes affected the cognitive decline in patients with CAS.

**Methods:**

The brain morphological differences were analyzed using surface and voxel-based morphometry, and the seed-based whole-brain functional connectivity (FC) abnormalities were analyzed using resting-state functional magnetic resonance imaging. Further, mediation analyses were performed to determine whether and how morphological and FC changes affect cognition in CAS patients.

**Results:**

The CAS-MCI (CAS patients with mild cognitive impairment) group performed worse in working memory, verbal fluency, and executive time. Cortical thickness (CT) of the left postcentral and superiorparietal were significantly reduced in CAS-MCI patients. The gray matter volume (GMV) of the right olfactory, left temporal pole (superior temporal gyrus) (TPOsup.L), left middle temporal gyrus (MTG.L), and left insula (INS.L) were decreased in the CAS-MCI group. Besides, decreased seed-based FC between TPOsup.L and left precuneus, between MTG.L and TPOsup.L, and between INS.L and MTG.L, left middle frontal gyrus, as well as Superior frontal gyrus, were found in CAS-MCI patients. Mediation analyses demonstrated that morphological and functional abnormalities fully mediated the association between the maximum degree of carotid stenosis and cognitive function.

**Conclusion:**

Multiple brain regions have decreased GMV and CT in CAS-MCI patients, along with disrupted seed-based FC. These morphological and functional changes play a crucial role in the cognitive impairment in CAS patients.

## 1 Introduction

Epidemiological studies have shown that 5% of people over 60 years of age have dementia, and 20% of patients with dementia are caused by atherosclerosis or other occlusive diseases affecting the cerebral vasculature (vascular dementia, VD) (Naylor et al., 2023). In recent years, the prevalence of cerebrovascular disease and dementia in China has shown an increasing trend (Yin et al., 2024). VD has become the second most common type of dementia after Alzheimer's disease (AD) (Jia et al., 2014). Increasing evidence suggests that carotid atherosclerotic stenosis (CAS) is an essential cause of vascular cognitive impairment (VCI), which can also lead to VD (Nie et al., 2018; Xu et al., 2022). However, whether and how CAS affects cognition remains indistinct (Paraskevas et al., 2021).

Asymptomatic CAS is related to cognitive impairment regardless of recognized vascular risk factors for VCI (Lal et al., 2017). Many possible underlying mechanisms have been proposed, such as silent microinfarctions and decreased cerebrovascular reserve due to hemodynamic changes (Paraskevas et al., 2021). Many studies have attempted to elucidate the “*in vivo*” mechanism of cognitive impairment observed in patients using novel imaging modalities such as resting-state functional magnetic resonance imaging (rs-fMRI) studies (Dutra, 2012; Huang et al., 2018; Chinda et al., 2022). Patients with external CAS exhibited worse cognitive function and lower whole-brain mean fractional anisotropy values based on diffusion tensor imaging compared to healthy controls (Porcu et al., 2020). These findings suggest generalized white matter degeneration. Gray matter atrophy is also increasingly being reported in patients with CAS (Gao et al., 2021; Wang P. et al., 2021; Duan et al., 2023; Liu et al., 2024). Besides, functional connectivity or network abnormalities have also been found in patients with CAS (Ren et al., 2022; Fan et al., 2024). Reduced connectivity in the default mode network (DMN) was found based on rs-fMRI (Porcu et al., 2020). Although brain morphological and functional changes were detected in CAS patients, the evidence could not unequivocally demonstrate the etiological role of CAS in cognitive decline (Naylor et al., 2023).

Thus, this study aims to investigate if the cognitive impairment in patients with CAS was related to any alterations in brain morphology and functional connectivity (FC). We aimed to analyze the changes in both morphological and functional features of the brain and their impact on cognitive decline among CAS patients. We hypothesized that brain morphological and function changes mediate the cognitive decline in CAS patients. To evaluate our hypotheses, we investigated CAS patients with mild cognitive impairment (MCI) using surface/voxel-based morphometry and resting-state FC analyses and compared them with CAS patients with normal cognition. The relationship between the morphological and functional abnormalities, global cognition, and maximum degree of carotid stenosis (CASmax) in patients was analyzed.

## 2 Material and methods

### 2.1 Study participants

The study subjects were recruited consecutively from a sample of patients from September 2021 to December 2022 at the Department of Vascular Surgery, Nanjing Drum Tower Hospital, the Affiliated Hospital of Nanjing University, Nanjing, China.

**Figure 1:**
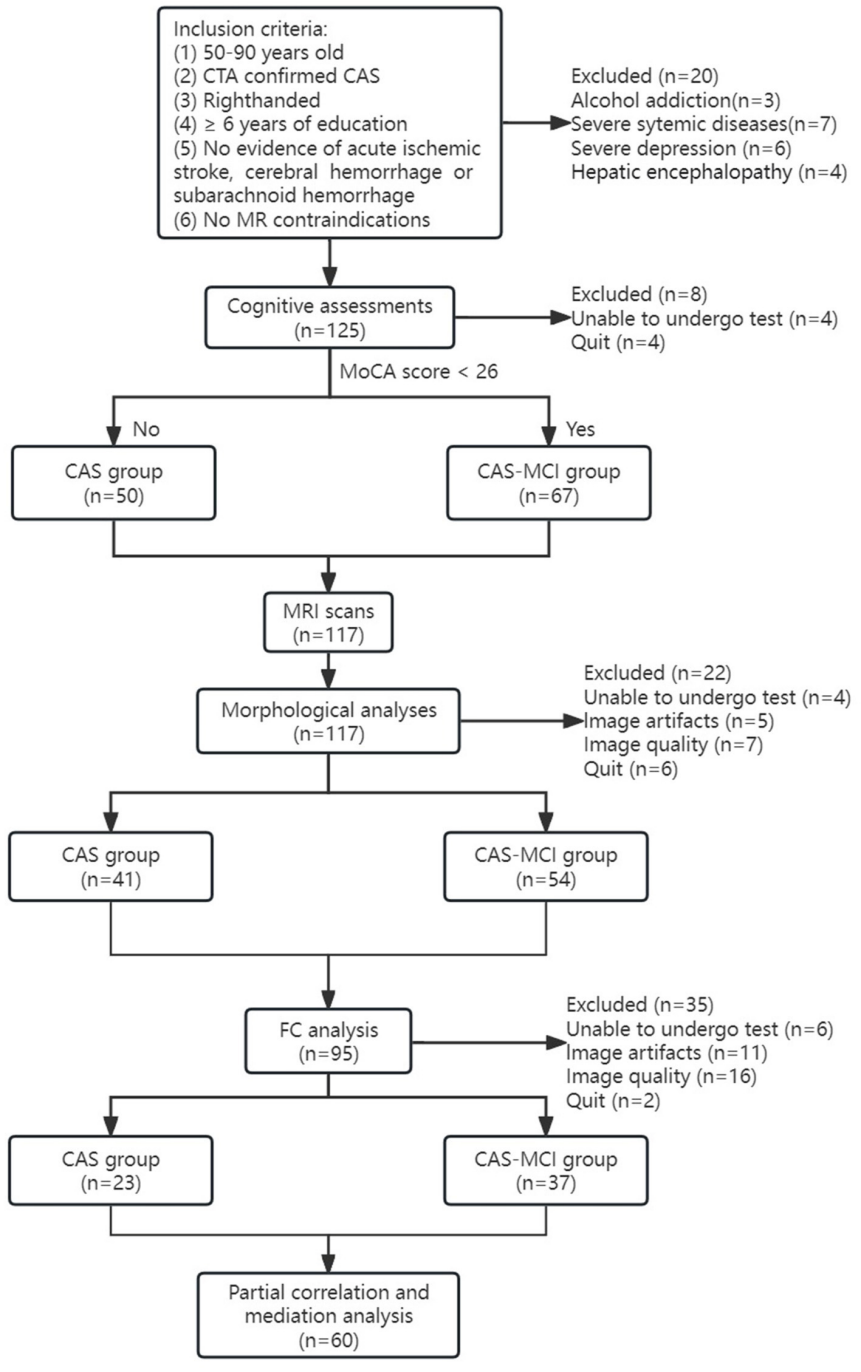
Flowchart of participants. CAS, carotid atherosclerotic stenosis; MCI, mild cognitive impairment; MRI, magnetic resonance imaging; FC, functional connectivity.

A total of 145 CAS patients were enrolled in this study. All participants underwent carotid computed tomography angiography (CTA). The degree of stenosis was calculated according to the North American Symptomatic Carotid Endarterectomy Trial (NASCET) criteria, and the degree of stenosis was divided into four levels: mild stenosis (0–49%), moderate stenosis (50–69%), severe stenosis (70–99%), and complete occlusion. Inclusion criteria and patient selection flowchart can be seen in Figure 1. Exclusion criteria: (1) severe systemic diseases such as congestive heart failure, infections, kidney disease, or malignancy; (2) Vascular dementia, Parkinson's disease, AD, epilepsy, mass, central nervous system infection, and other neurological diseases related cognitive decline; (3) Patients with severe depression, schizophrenia, and other mental and psychological diseases; (4) History of drug and alcohol addiction; (5) Other diseases that may affect cognitive function. In all 145 patients, 20 were excluded because of the exclusion criteria, 26 for unwillingness or inability to undergo cognitive assessment or MRI scanning or quit the study, and 39 because of image artifacts or poor image quality. Finally, a total of 117 patients underwent the MRI scan, of whom 60 were included in the final statistical analyses (Figure 1).

All participants were aware of the study protocol, willing to participate in this study, and signed the informed consent form. The study was approved by the Ethics Committee of Nanjing Drum Tower Hospital, the Affiliated Hospital of Nanjing University (AF/SC-08/03.0, 2021-368-02), and registered in the Chinese Clinical Trial Registry at chictr.org.cn (ChiCTR2200055544).

### 2.2 Clinical information, biochemical variables, and cognitive assessment

Demographic data, physical examination, medical history, and living habits of all participants were collected. Demographic data include sex, age, and years of education. Physical examination includes blood pressure and body mass index (BMI). Medical histories were recorded on the presence of stroke, hypertension, and diabetes mellitus. Living habits include smoking and alcohol consumption. Neuropsychological assessments were completed within 7 days of MRI scanning by a trained neuropsychologist blinded to the clinical diagnosis. The assessments included the Montreal cognitive assessment (MoCA), evaluation of working memory [digit span test-forward (DST-F) and backward (DST-B)], verbal fluency [animal naming test (ANT) and Boston naming test (BNT)], processing time [trail-making test A (TMT-A) and B (TMT-B)], and executive time [Stroop color and word test (SCWT)].

### 2.3 MRI acquisition

MRI scans were performed using a 3.0T scanner (Achieva TX, Philips, Netherlands) with an 8-channel head coil. High-resolution three-dimensional acceleration-field echo T1-weighted imaging was used to obtain whole-brain structural images. Scanning parameters: repetition time or time of repetition (TR) = 9.9 ms, echo time or time of echo (TE) = 4.6 ms, flip angle = 8°, resolution = 1 × 1 × 1 mm, field of view (FOV) = 256 × 256 mm. The fMRI data were acquired by a gradient-echo planar imaging sequence with parameters: repetition time, 2,000 ms, echo time, 30 ms; flip angle, 90°; field of view, 192 × 192 mm; and slice thickness, 4 mm; gap, 0 mm; 35 interleaved axial slices; voxel size, 3 × 3 × 4 mm; and 230 volumes. Subjects had their heads immobilized with sponges, and rubber earplugs in their ears, and were instructed to keep their heads as still as possible.

### 2.4 Image analyses

Structural MRI data processing was performed using the Computational Anatomy Toolbox (CAT12, https://neuro-jena.github.io/cat/) together with Statistics Parametric Mapping version 12 (SPM12, http://www.fil.ion.ucl.ac.uk/spm/software/spm12) implemented in MATLAB R2021b (MathWorks, Natick, MA, USA) for gray matter volume (GMV) and cortical thickness (CT) estimation.

Surface-based morphometry (SBM) was used for CT analysis. Cat12 automatically evaluates CT and the central surface in one step using the Projection Based Thickness (PBT) method. Voxel-based morphometry (VBM) was used for GMV analysis also using Cat12. The data preprocessing includes automatic segmentation of gray matter (GM), white matter (WM), and cerebrospinal fluid (CSF), affine registration to the Montreal Neurological Institute (MNI) template space, and non-linear deformation. A 12-mm full-width at half-maximum (FWHM) Gaussian kernel was used to smooth surface data. GM images were smoothed with an 8-mm FWHM (Jenkinson et al., 2002). The GM, WM, and CSF partitions were summarized as the total intracranial volume (TIV), which was calculated to adjust for head size differences.

Rs-fMRI data preprocessing was performed using the DPABI package (http://rfmri.org/dpabi) (Yan et al., 2016). The preprocessing process consisted of: (1) slice timing; (2) realigning and limiting head motion to translation < 3 mm and rotation < 3°; (3) normalization to MNI space; (4) smooth with FWHM [6 6 6] Gaussian kernel and linear detrend; (5) regressing out nuisance covariates: WM and CSF signals; (6) filtering with retention of signals at 0.01 Hz < f < 0.08 Hz.

GMV differential clusters based on VBM analysis were selected as seed regions for seed-based whole-brain FC analysis. All processing was performed using the DPABI package. The steps included: extracting the average time series of oximeter-dependent signals within the seed regions, calculating the correlation between this time series and other voxel time series in the whole brain, and finally obtaining the functional connectivity map of the whole brain. Correlation coefficients were Fisher *r*-to-*z* transformed to normalize. Multiple comparison correction was performed using the Gaussian random field (GRF) with associated Bonferroni correction being performed with a voxel-wise *P*-value < 0.001 and a cluster-wise *P*-value < 0.05.

### 2.5 Statistical analyses

All statistical analyses were performed using IBM SPSS 26.0 software (IBM, Armonk, New York). Continuous variables were first tested for Kolmogorov-Smirnov normality, variables that conform to the normal distribution are expressed as means ($\bar{x}$) ± standard deviations (*s*), and independent two-sample *t*-test were used to compare variables; those that do not conform to the normal distribution expressed as median [inter-quartile range, *M (Q1-Q3)*], and Mann-Whitney *U*-test was used to compare variables. The comparison of categorical variables was conducted using either the chi-square test or Fisher's exact test. Comparisons between groups were performed using a general linear model, and partial correlation analysis was used to determine the correlation between GMV/CT/FC differences and cognitive scores. Individual cognitive domain scores from each cognitive assessment were converted to standardized *z*-scores (Zhang et al., 2020). Age, sex, education years, hypertension, diabetes mellitus, and TIV were included in the analysis as covariates. Additionally, the Benjamini–Hochberg false discovery rate (FDR) correction was applied to reduce the inflation of type 1 error from multiple comparisons. The PROCESS SPSS toolbox (Hayes, 2012) was used to perform mediation analysis. The indirect effect in the model was estimated using 5,000 bias-corrected bootstraps with a 95% confidence interval (CI). A *p*-value < 0.05 was considered significant.

CT and GMV analyses were performed using two-sample *t*-tests on SPM12. Age, sex, education years, hypertension, diabetes mellitus, and TIV were included as covariates. Family-wise error (FWE) method was conducted for multiple comparisons at a threshold of voxel-level *P* < 0.001, and cluster-level *P* < 0.05. These procedures were implemented to ensure the validity and reliability of the results.

Rs-fMRI Data were statistically analyzed using the DPABI package (Yan et al., 2016) and differences between groups were analyzed using independent two-sample *t*-tests. Correction for multiple comparisons was performed using GRF correction with a corrected threshold of *P* < 0.05 at the cluster level and *P* < 0.01 at the voxel level.

For visualization of the results, XjView (Xu Cui, Human Neuroimaging Lab, Baylor College of Medicine; http://www.alivelearn.net/xjview/) and BrainNet Viewer (http://www.nitrc.org/projects/bnv/) were utilized.

## 3 Results

### 3.1 Clinical and cognitive characteristics of participants

A total of 117 patients were included in the analysis, of whom 50 (42.7%) were CAS patients and 67 (57.3%) were CAS-MCI patients. As shown in Table 1, there were no significant differences between groups in gender, age, BMI, blood pressure, stroke, diabetes mellitus, smoking history, and alcohol consumption (both *P* > 0.05). Patients with MCI had higher degree of stenosis (*P* = 0.042) and lower years of education (*P* = 0.004). Patients with MCI were more likely to have hypertension (*P* = 0.004). The CAS-MCI group performed worse in working memory, verbal fluency [ANT showed a quasi-significant level (*P* = 0.052)], and executive time (both *P* < 0.05) while there was no significant difference found in processing time (both *P* > 0.05).

**Table 1:** Characteristics and cognition assessment scores of CAS patients.

	**Total (*n* = 117)**	**CAS group (*n* = 50)**	**CAS-MCI group (*n* = 67)**	***P***
Sex, male (%)	90 (76.90)	38 (76.00)	52 (77.4)	0.697
Age (x ± s, y)	66.47 ± 7.22	64.17 ± 7.37	67.89 ± 6.84	0.052
Maximum degree of carotid stenosis (CTA) [M (Q1–Q3), %]	85 (60–85)	25 (25–85)	85 (60–85)	0.042
BMI [M (Q1–Q3), kg/m^2^]	24.22 (22.04–25.50)	24.12 (22.04–26.22)	24.45 (22.20–25.48)	0.915
Education [M (Q1–Q3), y]	10.50 (8.25–12)	12 (11–13)	9 (6–12)	0.004
Systolic BP (x ± s, mmHg)	135.48 ± 16.96	132.91 ± 15.46	136.97 ± 17.42	0.364
Diastolic BP (x ± s, mmHg)	76.66 ± 12.18	77.96 ± 8.08	75.68 ± 13.93	0.426
Stroke (*n*, %)	55 (47.01)	24 (48.00)	31 (46.20)	0.605
Hypertension (*n*, %)	80 (68.38)	29 (58.00)	51 (76.12)	0.004
Diabetes mellitus (*n*, %)	45 (38.46)	20 (40.00)	25 (37.31)	0.07
Smoking history (*n*, %)	64 (54.70)	28 (56.00)	36 (53.73)	0.438
Alcohol consumption (*n*, %)	55 (47.01)	21 (42.00)	34 (50.75)	0.605
**Working memory**				
DST-F	8 (7–9)	9 (8–9)	7 (7–8)	< 0.001
DST-B	5 (4–5)	5 (4–5)	4 (3–5)	0.003
**Verbal fluency**				
ANT	17 ± 6	21 ± 4	14 ± 5	0.052
BNT	38 (28–48)	44 (37–48)	29 (27–30)	0.013
**Processing time**				
TMT-A	68 (53–89)	63 (50–87)	72 (59–90)	0.136
TMT-B	167 ± 49	156 ± 43	174 ± 51	0.166
**Executive time**				
SCWT-C	23 (18–31)	20 (15–29)	24 (21–35)	0.027
SCWT-W	30 (22–37)	25 (18–33)	31 (25–38)	0.015
SCWT-CW	49 (36–67)	37 (27–48)	60 (42–77)	< 0.001

CAS, carotid atherosclerotic stenosis; MCI, mild cognitive impairment; CTA, computed tomography angiology; BMI, body mass index; BP, blood pressure; DST-F, digit span test-forward; DST-B, digit span test-backward; ANT, animal naming test; BNT, Boston naming test; TMA-A, trail-making test A; TMT-B, trail-making test B; SCWT-C, Stroop color and word test-color; SCWT-W, Stroop color and word test-word; SCWT-CW, Stroop color and word test-color and word.

### 3.2 Morphological changes

**Figure 2:**
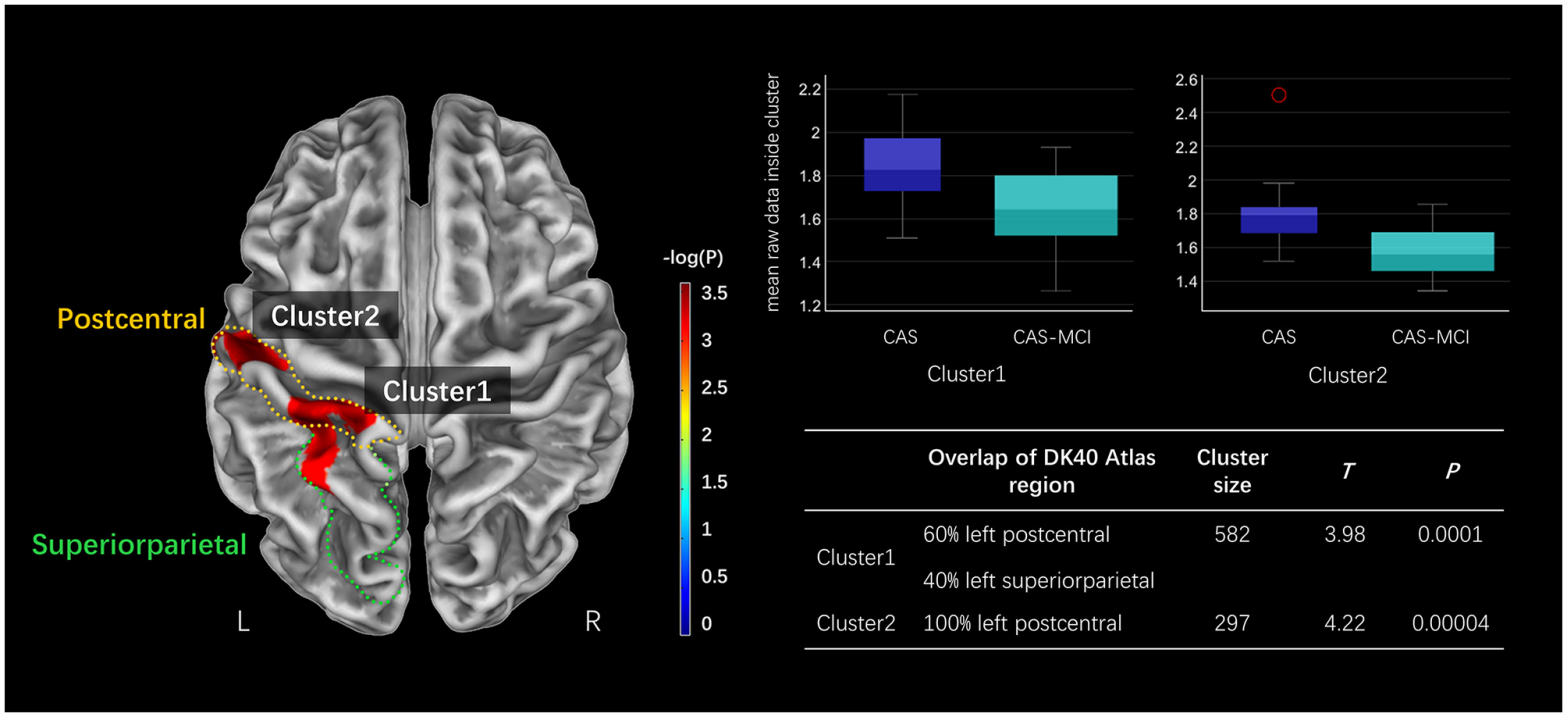
Clusters significantly decreased in the CAS-MCI group compared to the CAS group in cortical thickness based on SBM analysis. The image shows the result of the two-sample *t*-test comparing the cortical thickness of the CAS-MCI patients with CAS patients without cognitive impairment (contrast “CAS-MCI group < CAS group”), at a threshold of voxel-level *P* < 0.001, cluster-level *P* < 0.05 FWE corrected. The brain regions of differential clusters overlapping on the DK40 atlas were outlined in yellow and green. SBM, surface-based morphometry; CAS, carotid atherosclerotic stenosis; MCI, mild cognitive impairment; FWE, family-wise error; DK40 Atlas, Desikan-Killiany atlas.

Figure 2 presents the CT results obtained from the SBM analysis. There were two clusters found significantly reduced in CAS-MCI patients compared to CAS patients after correction for age, sex, education years, hypertension, diabetes mellitus, and TIV. The differences were mainly concentrated in left postcentral and left superiorparietal [Cluster1: 60% left postcentral and 40% left superiorparietal overlap on Desikan-Killiany (DK40) atlas; Cluster2: 100% left postcentral overlap on DK40 atlas] (at a threshold of voxel-level *P* < 0.001, cluster-level *P* < 0.05 FWE corrected).

**Figure 3:**
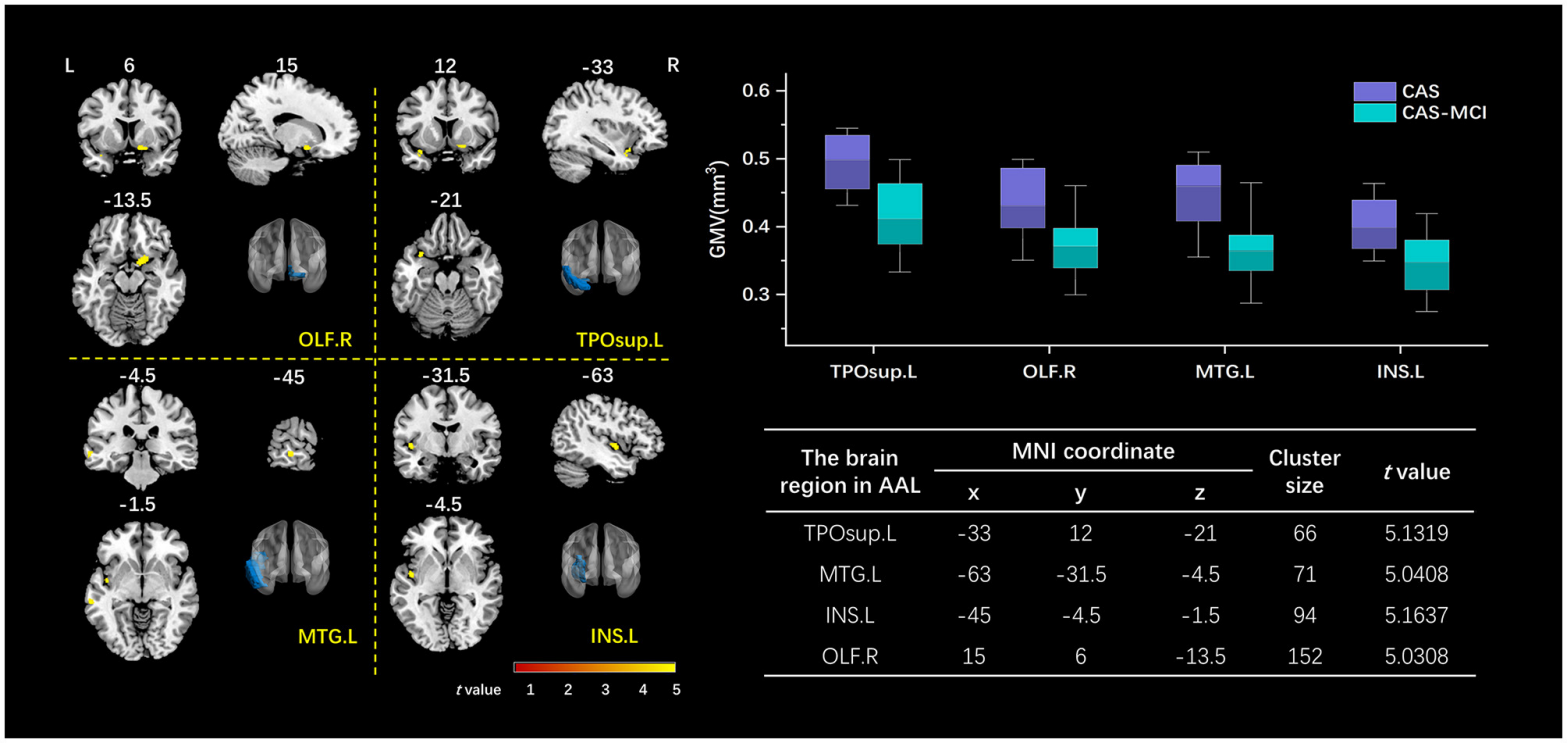
Clusters with a significant volumetric difference revealed by VBM analysis. The volume of OLF.R, TPOsup.L, MTG.L, and INS.L decreased in the CAS-MCI group compared to the CAS group. The image shows the result of the two-sample *t*-test comparing the gray matter value of the CAS-MCI patients with CAS patients without cognitive impairment (contrast “CAS-MCI group < CAS group”), at a threshold of *P* < 0.05 FWE corrected. The brain regions of differential clusters were marked in blue. x, y, z are the *t* value peek voxel coordinates in MNI space. VBM, voxel-based morphometry; TPOsup.L, left temporal pole: superior temporal gyrus; MTG.L, left middle temporal gyrus; INS.L, left insula; OLF.R, right olfactory; CAS, carotid atherosclerotic stenosis; MCI, mild cognitive impairment; FWE, family-wise error; MNI, Montreal Neurological Institute.

As shown in Figure 3, patients with MCI show significantly decreased GMV in the right olfactory (OLF.R), left temporal pole (superior temporal gyrus) (TPOsup.L), left middle temporal gyrus (MTG.L), and left insula (INS.L) after correction for age, sex, education years, hypertension, diabetes mellitus, and TIV (FWE corrected, cluster-level *P* < 0.05).

### 3.3 Functional connectivity changes

**Figure 4:**
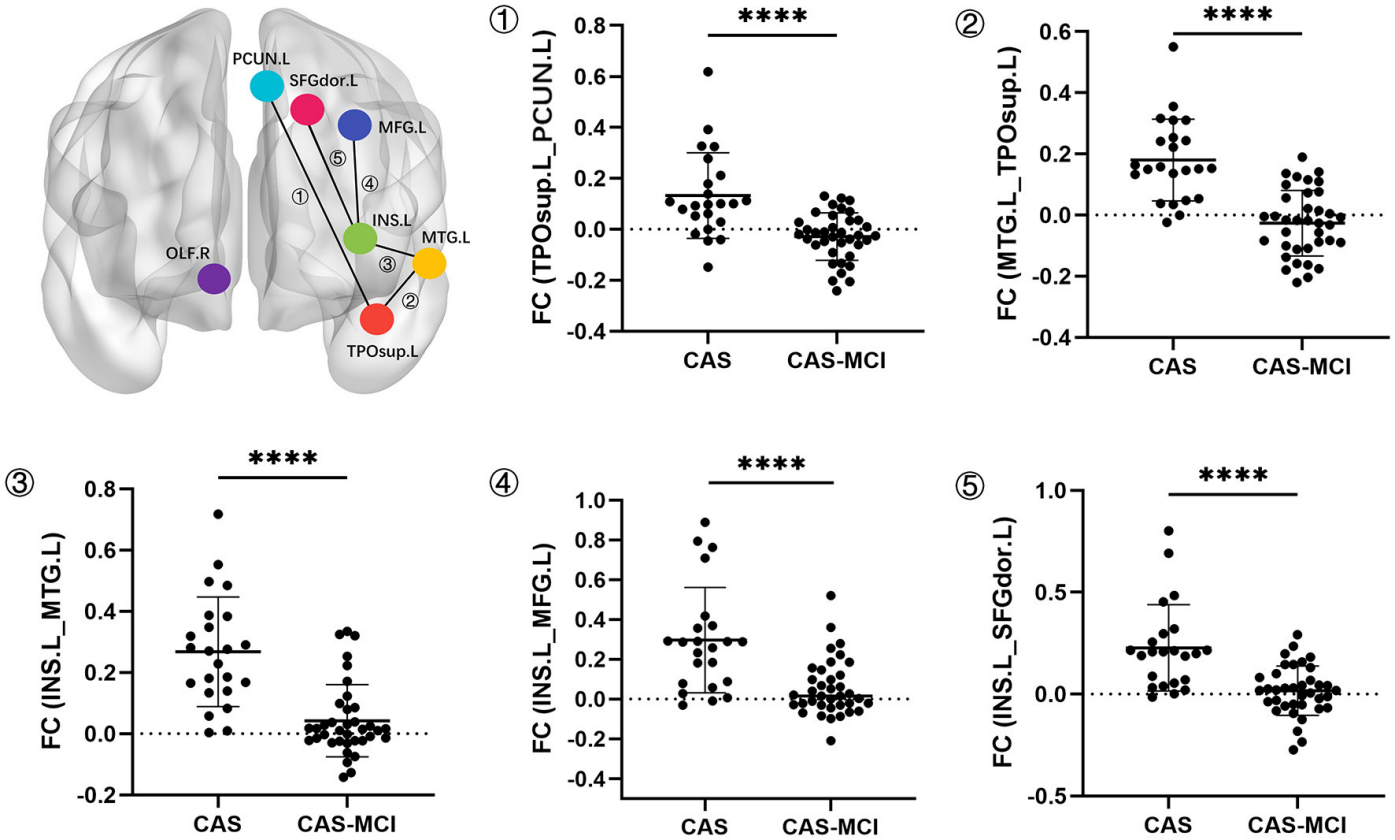
Seed-based whole brain functional connectivity decreased clusters in the CAS-MCI group compared to the CAS group. ^****^ GRF correction, voxel *P* < 0.001, cluster *P* < 0.05. CAS, carotid atherosclerotic stenosis; MCI, mild cognitive impairment; FC, functional connectivity; TPOsup.L, left temporal pole: superior temporal gyrus; OLF.R, right olfactory; MTG.L, left middle temporal gyrus; INS.L, left insula; PCUN.L, left precuneus; MFG.L, left middle frontal gyrus; SFGdor.L, Superior frontal gyrus (dorsolateral).

The results of seed-based FC changes are shown in Figure 4. With TPOsup.L and MTG.L as seed regions, abnormally decreased FC were found in the left precuneus (PCUN.L) (cluster size: 30, *t* = 4.5864) and TPOsup.L (cluster size: 49, *t* = 5.7589) in CAS-MCI patients, respectively. With the seed placed in INS.L, CAS-MCI patients showed significantly lower FC with the MTG.L (cluster size: 222, *t* = 4.7572), left middle frontal gyrus (MFG.L) (cluster size: 110, *t* = 4.9181), as well as Superior frontal gyrus dorsolateral (SFGdor.L) (cluster size: 147, *t* = 5.1656) (all GRF corrected, voxel *P* < 0.001, cluster *P* < 0.05). These results indicated that CAS-MCI patients had disrupted seed-based functional connectivity compared with CAS patients.

### 3.4 Partial correlation

**Figure 5:**
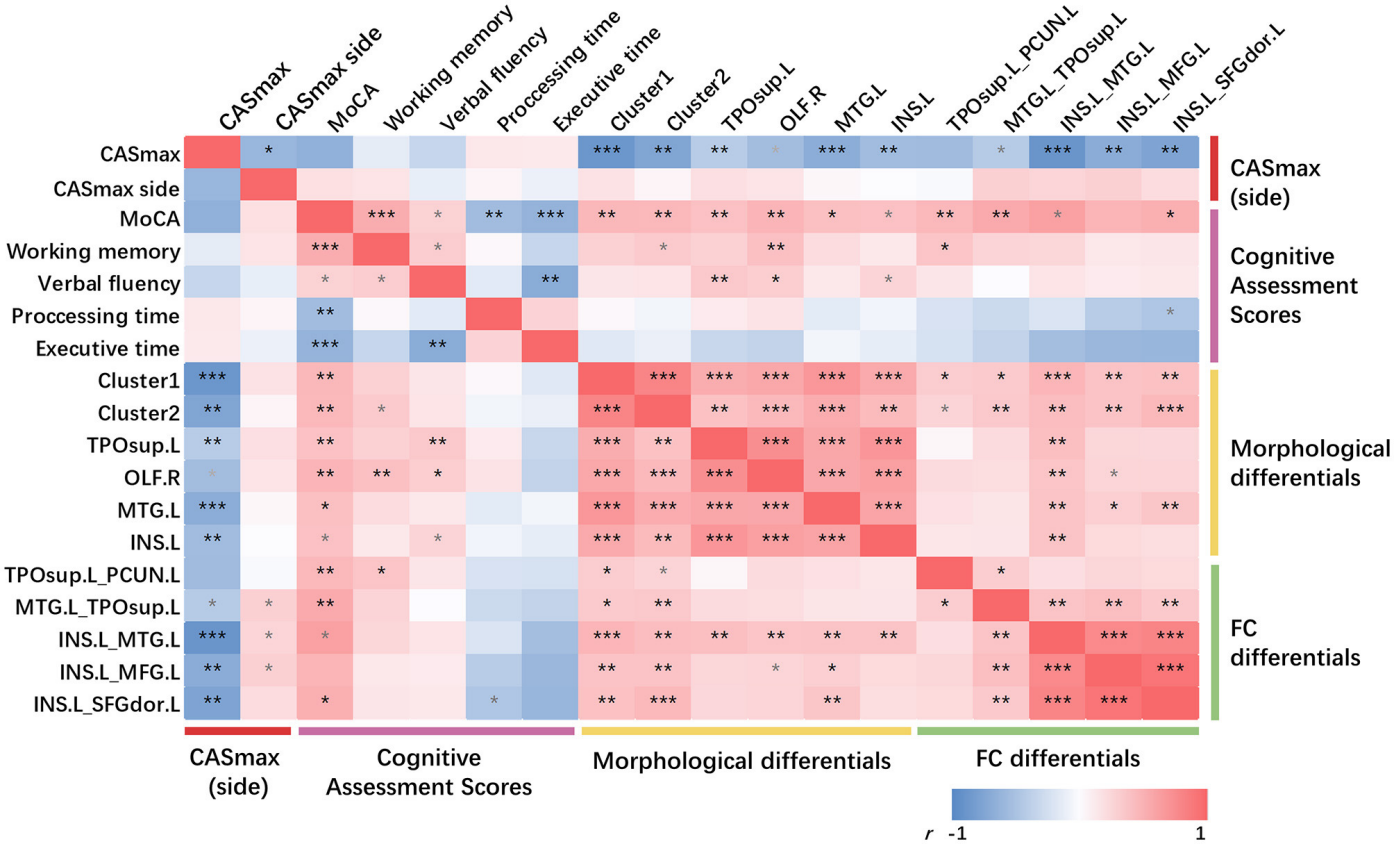
Partial correlations among CASmax, GMV changes, functional connectivity changes, and cognitive performance. Partial correlation analysis with age, sex, education, hypertension, and diabetes mellitus controlled. *p < 0.05, **p < 0.01, ***p < 0.001, FDR corrected, *p*-values did not survive after FDR correction were marked in gray. Positive correlations are marked in red, negative correlations in blue. Dark red represents the maximum degree of carotid stenosis, peachblow is cognitive performance, yellow is GMV changes and green is functional connectivity changes. Cluster1, 60% left postcentral and 40% superiorparietal overlap on DK40 Atlas; Cluster2, 100% left postcentral on DK40 Atlas. MoCA, Montreal cognitive assessment; CASmax, the maximum degree of carotid atherosclerotic stenosis; GMV, gray matter volume; TPOsup.L, left temporal pole: superior temporal gyrus; OLF.R, right olfactory; MTG.L, left middle temporal gyrus; INS.L, left insula; PCUN.L, left precuneus; MFG.L, left middle frontal gyrus; SFGdor.L, Superior frontal gyrus (dorsolateral).

As can be seen in Figure 5, partial correlation analysis controlling age, sex, education, hypertension, and diabetes mellitus showed that most of the morphological differential clusters and FC changes were significantly negatively correlated with CASmax (10 in 11, both *P* < 0.05), while being positively correlated with the MoCA score (10 in 11, both *P* < 0.05). The majority of the observed correlations remain significant even after undergoing FDR correction. Further analysis between the side of CASmax, cognitive domains, and imaging changes showed positive correlation between the side of CASmax and FC changes (3 in 5, both *P* < 0.05), but failed to be corrected for FDR correction.

No significant correlation was found between the cognitive domain and CASmax. Only little correlations were detected between the cognitive domains and morphological or FC changes.

There were significant positive correlations between FC and CT changes (both *P* < 0.05). Significant positive correlations were found between all GMV changes and FC between INS.L and MTG.L (both *P* < 0.05). MTG.L positively correlated with FC changes when using INS.L as seed regions (both *P* < 0.05).

### 3.5 Mediation analysis

**Figure 6:**
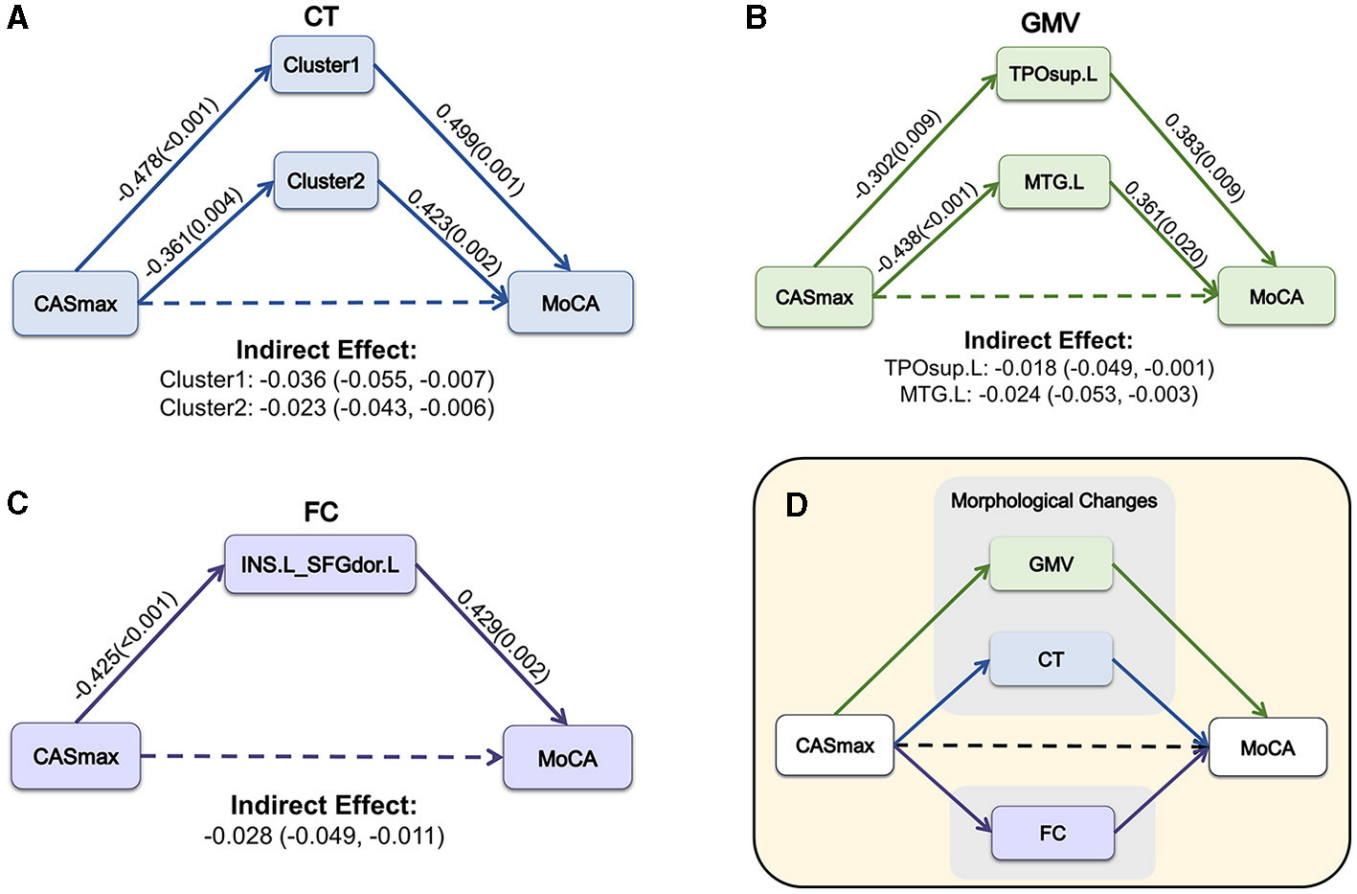
The mediation model among CASmax, morphological and FC changes, and global cognition in CAS patients. Associations of CASmax, the mediator of CT changes, and MoCA scores **(A)**. Associations of CASmax, the mediator of GMV changes, and MoCA scores **(B)**. Associations of CASmax, the mediator of FC changes, and MoCA scores **(C)**. These mediation models indicate that morphological and FC changes mediated cognitive function in CAS patients **(D)**. Standardized β-coefficient was derived from mediation models controlling for age, sex, education, hypertension, and diabetes mellitus. Values are standardized path coefficients with *P*-values or 95% CIs in parentheses. The effect of CASmax on cognition was found to be fully mediated by differential clusters. Cluster1, 60% left postcentral and 40% superiorparietal overlap on DK40 Atlas; Cluster2, 100% left postcentral on DK40 Atlas. CASmax, the maximum degree of carotid atherosclerotic stenosis; MoCA, Montreal cognitive assessment; CT, cortical thickness; GMV, gray matter volume; TPOsup.L, left temporal pole: superior temporal gyrus; MTG.L, left middle temporal gyrus; FC, functional connectivity; INS.L, left insula; SFGdor.L, Superior frontal gyrus (dorsolateral); DK40 Atlas, Desikan-Killiany atlas.

Mediation analyses were performed to determine whether morphological and FC changes served as a mediator between CASmax and cognitive impairment. Figure 6A demonstrates that the association between CASmax and MoCA scores was fully mediated by CT of cluster1 [β = −0.036, 95% bootstrap CI (−0.055, −0.007)], cluster2 [β = −0.023, 95% bootstrap CI (−0.043, −0.006)]. GMV of TPOsup.L [β = −0.018, 95% bootstrap CI (−0.049, −0.001)], and MTG.L [β = −0.024, 95% bootstrap CI (−0.053, −0.003)] also fully mediated the association between CASmax and MoCA scores (Figure 6B). Meanwhile, FC between INS.L and SFGdor.L was found to fully mediate the correlation of CASmax and MoCA scores [β = −0.028, 95% bootstrap CI (−0.049, −0.011)] (Figure 6C). Taken together, morphological and FC changes showed mediating effects on the relationships of CASmax and cognition (Figure 6D).

## 4 Discussion

In this study, we examined brain morphological and functional changes in patients with CAS-MCI, using CAS patients as the control group. We found significant CT (left postcentral and superiorparietal) and GMV (TPOsup.L, MTG.L, INS.L, and OLF.L) decrease in CAS patients with MCI. With INS.L as a seed region, CAS-MCI patients showed decreased FC in MTG.L, MFG.L, and SFGdor.L. TPOsup.L-based FC also showed that CAS-MCI patients had abnormally decreased FC in PCUN.L compared with CAS patients. The same seed-based FC decrement was detected between MTG.L and TPOsup.L using MTG.L as a seed region. We found significant negative correlations between CASmax and both morphological differential clusters and FC, while significant positive correlations were observed between these changes and the MoCA score. By establishing mediation analyses, we found CASmax can affect cognitive function via morphological and FC changes.

VD has received increasing attention in recent years, and the impact of CAS on cognitive function has also drawn a lot of interest from clinicians. We found that there were between-group differences in CASmax, years of education, and blood pressure between CAS and CAS-MCI patients, which is consistent with the results of previous studies (Paraskevas et al., 2021). Studies have demonstrated that patients with higher stenosis and lower years of education are more likely to show impaired cognitive function (Paraskevas et al., 2021). We therefore strictly controlled the years of education, and hypertension in our subsequent analyses. We also included gender, age, and diabetes mellitus, which may have an impact on cognition, as covariates in our subsequent analyses to ensure the reliability of the results.

Cortical thinning and brain atrophy, as important imaging biomarkers in various brain diseases, were reported in previous studies (Hwang et al., 2015; Lambert et al., 2015; Nickel et al., 2019; Duan et al., 2023). We found left postcentral and superior parietal had significant CT decrement in CAS-MCI patients and the CT changes were associated with CASmax, MoCA scores, GMV changes, and seed-based FC changes. There are similarities between these results and previous studies showing cortical atrophy in CAS or MCI patients in multiple brain regions such as left precuneus, right anterior cingulate, right cingulate gyrus, bilateral somatosensory/motor, fusiform, hippocampus, and left lateral temporal areas (Singh et al., 2006; Seo et al., 2010; Marshall et al., 2017; Ren et al., 2022; Dong et al., 2023; Ghaznawi et al., 2023). Furthermore, studies have confirmed that cortical atrophy can reach 3.1–3.7% in carotid occlusion patients (Asllani et al., 2016; Wang P. et al., 2021). However, our results contradict the previous research that has suggested although CAS patients had lower CT in the middle cerebral artery (MCA) region on the side of carotid stenosis, there was no correlation between the degree of stenosis, cortical thickness, and cognition (Nickel et al., 2019). Left postcentral and superior parietal have been implicated in the attentional network (Xuan et al., 2016). These regional CT changes and the results of the correlation analyses suggest that the attentional network is impaired in CAS-MCI patients. This is consistent with the results of the cognitive assessment scores. Our findings indicate that no significant CT changes were detected in the hippocampus, temporal lobe, cingulate, fusiform, or precuneus regions. Although we did not find many CT differences as in previous studies, we did find significant decrements in the GMV of TPOsup.L, MTG.L, INS.L, and OLF.R in CAS-MCI patients. The possible explanation for these abnormal CT and GMV decrements may refer to hypoperfusion. Mouse model (Nyitrai et al., 2018) and human MRI studies (Marshall et al., 2017, 2023) both confirmed the correlation between the hypoperfusion caused by CAS or carotid occlusion and cortical atrophy. The temporal lobe atrophy was reported not only in AD but also in PD and MCI patients (Hall and Lewis, 2019; Wang E. et al., 2021). The temporal lobe and INS are the first cortical regions to be affected by Lewy bodies (Braak et al., 2003). Meanwhile, OLF atrophy was reported in early-stage AD-related MCI (Fjaeldstad et al., 2017; Murphy, 2019; Chen et al., 2022). In agreement with previous studies, partial correlation analyses showed that GMV changes are associated with impaired global cognition.

It's worth noting that with TPOsup.L and MTG.L as seed regions, abnormally decreased FC were found in PCUN.L and TPOsup.L, respectively. With INS.L as a seed region, abnormally decreased FC were found in MTG.L, MFG.L, and SFG.L. These regions are all important in the default mode network (DMN) (Qi et al., 2010; Herlin et al., 2021; Lu et al., 2022). Considerable research have demonstrated a significant reduction in DMN activation and FC in individuals with neurodegeneration diseases, same changes also were found in MCI patients (Wang et al., 2017; Eyler et al., 2019; Gao et al., 2019; Yuan et al., 2021; Wei et al., 2022). Our findings in FC changes are in line with those previous studies. The higher-order system, including DMN, appears to be last impacted compared to the lower-order cognitive process (Gao et al., 2019). However, decreased FC in DMN may be a precursor to VCI (Gao et al., 2019). Interestingly, a study shows compensatory increases in FC in MCI patients (Qi et al., 2010), this was not found in our study. Hemispheric detection suggests that cerebral hemispheres ipsilateral were mainly decreased while contralateral increased, this may be an important mechanism that maintains clinical asymptomatic performance in CAS patients (Huang et al., 2018; He et al., 2020). In this study, partial correlation analyses showed that the side of CASmax was not significantly correlated with FC changes, while FC changes were negatively correlated with CASmax. Most of the FC changes are positively related to the global cognition decline. This observation may support the hypothesis that DMN abnormality in CAS-MCI patients was not only caused by MCI but also closely related to the severity of CAS, regardless of the side of CASmax. These findings and previous studies suggest that the brain morphological and functional changes in CAS-MCI are somewhat similar to those in neurodegenerative diseases. This has important implications for the understanding of vascular and non-vascular cognitive impairment.

The most important finding of our study was the mediating effect of morphological and functional changes between CASmax and cognition. The correlation between CAS and cognition is complicated and remains unclear. Risk factors for vascular diseases are also related to cognitive function, which makes it difficult to figure out the role CAS plays in cognitive impairment (Paraskevas et al., 2021). To the best of our knowledge, no study has examined whether and how brain morphological and functional changes affect cognition in CAS patients with MCI at the same time. It is currently believed that hemodynamic abnormality is an important reason that may lead to brain morphological changes in CAS patients eventually causing cognitive impairment (Paraskevas et al., 2021; Duan et al., 2023). The correlation between high-grade CAS and cognitive impairment is related to silent embolization and hypoperfusion. However, there is no conclusive proof that CAS directly caused cognitive impairment through silent infarction or by contributing to the pathophysiology of lacunar infarction or WM hyperintensities (Dutra, 2012; Paraskevas et al., 2021). Previous studies have found mediating effects between carotid intima-media thickness and cognition with GMV of special brain regions (such as the thalamus) or WM hyperintensity volume as mediators (Della-Morte et al., 2018; Zhang et al., 2020). Few studies determined whether brain atrophy mediates cognitive impairment in CAS patients. Our findings expand upon previous knowledge about the role of brain morphological changes in cognitive impairment in CAS patients. Besides, few studies have proved the association between FC and cognition in CAS patients (Huang et al., 2018; Gao et al., 2019; He et al., 2020). However, there was no direct evidence of an FC-mediated mediating effect between cognitive impairment and degree of stenosis in CAS patients in previous studies. This is complemented by our study. Our findings indicate that brain morphological changes together with functional changes may be a potential mechanism for CASmax to affect cognitive impairment. We also performed chain mediation analyses at the same time, but sadly, we were unable to determine the causal relationship between brain morphological and functional changes in CAS patients with cognitive impairment. These findings are important for understanding the potential implications of cognitive impairment in CAS patients.

This study had several limitations. First, this study is only a cross-sectional study, the effect of carotid revascularization on cognitive function or brain changes in patients remains unknown. As mentioned in the guidelines, whether carotid interventions improve cognitive function still lacks direct evidence (Naylor et al., 2023). This is an important issue for future research. Second, the correlation between brain morphological changes and FC changes remains unknown. The latest research suggests that the effect of brain structure on function may be greater than we expected (Pang et al., 2023). Thus, future studies are needed to further investigate whether brain morphological changes impact functional connectivity in CAS patients eventually causing cognitive impairment. Third, the sample size is relatively small because of our strict inclusion criteria. Besides, patients visiting our department are those who need carotid endarterectomy and/or carotid artery stenting, only a small percentage of CAS patients need carotid revascularization, making data collection more difficult. It is necessary to enlarge the sample size to obtain more accurate and comprehensive results.

## 5 Conclusion

This study demonstrates that CAS patients with MCI exhibit a reduction in the volume and cortical thickness of multiple brain regions. Meanwhile, decreased seed-based functional connectivity was also found in this study. These changes may collectively contribute to the development of cognitive impairment in CAS patients.

## Data availability statement

The data analyzed in this study is subject to the following licenses/restrictions: the data of this study are available from the corresponding author upon reasonable request. Requests to access these datasets should be directed to TQ, qiaotongmail@nju.edu.cn and WZ, njumed_zw@smail.nju.edu.cn.

## Ethics statement

The studies involving humans were approved by the Ethics Committee of Nanjing Drum Tower Hospital, the Affiliated Hospital of Nanjing University. The studies were conducted in accordance with the local legislation and institutional requirements. The participants provided their written informed consent to participate in this study.

## Author contributions

SM: Conceptualization, Formal analysis, Investigation, Methodology, Software, Writing—original draft. CT: Formal analysis, Investigation, Software, Validation, Visualization, Writing—original draft. CL: Data curation, Methodology, Resources, Supervision, Validation, Writing—review & editing. XL: Formal analysis, Investigation, Visualization, Writing—review & editing. ZC: Data curation, Formal analysis, Investigation, Writing—review & editing. JC: Investigation, Resources, Software, Writing—review & editing. ZL: Data curation, Investigation, Writing—review & editing. MZ: Investigation, Software, Writing—review & editing. BZ: Data curation, Investigation, Writing—review & editing. WZ: Conceptualization, Data curation, Methodology, Project administration, Resources, Software, Writing—review & editing. TQ: Writing—review & editing, Data curation, Funding acquisition, Methodology, Project administration, Resources, Supervision.
